# AMIGOS III: pseudo-torsion angle visualization and motif-based structure comparison of nucleic acids

**DOI:** 10.1093/bioinformatics/btac207

**Published:** 2022-04-06

**Authors:** Morgan Shine, Chengxin Zhang, Anna Marie Pyle

**Affiliations:** Yale Combined Program in the Biological and Biomedical Sciences, Yale University, New Haven, CT 06511, USA; Department of Molecular, Cellular and Developmental Biology, Yale University, New Haven, CT 06511, USA; Department of Chemistry, Yale University, New Haven, CT 06511, USA; Howard Hughes Medical Institute, Chevy Chase, MD 20815, USA; Department of Molecular, Cellular and Developmental Biology, Yale University, New Haven, CT 06511, USA; Department of Chemistry, Yale University, New Haven, CT 06511, USA; Howard Hughes Medical Institute, Chevy Chase, MD 20815, USA

## Abstract

**Motivation:**

The full description of nucleic acid conformation involves eight torsion angles per nucleotide. To simplify this description, we previously developed a representation of the nucleic acid backbone that assigns each nucleotide a pair of pseudo-torsion angles (eta and theta defined by P and C4ʹ atoms; or etaʹ and thetaʹ defined by P and C1ʹ atoms). A Java program, AMIGOS II, is currently available for calculating eta and theta angles for RNA and for performing motif searches based on eta and theta angles. However, AMIGOS II lacks the ability to parse DNA structures and to calculate etaʹ and thetaʹ angles. It also has little visualization capacity for 3D structure, making it difficult for users to interpret the computational results.

**Results:**

We present AMIGOS III, a PyMOL plugin that calculates the pseudo-torsion angles eta, theta, etaʹ and thetaʹ for both DNA and RNA structures and performs motif searching based on these angles. Compared to AMIGOS II, AMIGOS III offers improved pseudo-torsion angle visualization for RNA and faster nucleic acid worm database generation; it also introduces pseudo-torsion angle visualization for DNA and nucleic acid worm visualization. Its integration into PyMOL enables easy preparation of tertiary structure inputs and intuitive visualization of involved structures.

**Availability and implementation:**

https://github.com/pylelab/AMIGOSIII.

**Supplementary information:**

Supplementary data are available at *Bioinformatics* online.

## 1 Introduction

Nucleotide conformation is determined by six backbone torsion angles, the torsion angle of the bond between the base and the sugar, and the sugar pucker, making its description an eight-dimensional problem. To reduce this complexity, we previously developed an alternative representation in which each nucleotide is described by two pseudo-torsion angles: eta (C4ʹ_*i*__−__1_–P_*i*_–C4ʹ_*i*_–P_*i*__+1_) and theta (P_*i*_–C4ʹ_*i*_–P_*i*__+1_–C4ʹ_*i*__+1_) (Duarte and Pyle, 1998). Eta and theta angles can be plotted in an analogous manner to phi and psi angles in proteins, resulting in a Ramachandran-like plot (Nucleic Acid Ramachandran, or NARama) which facilitates quick and accurate categorization of nucleic acid structure (Duarte and Pyle, 1998; Wadley *et al.*, 2007).

This torsional space representation of nucleic acid tertiary structure has proven to be a powerful tool for structural analysis. It has been used to identify novel structural elements, or motifs (Adams *et al.*, 2004; Chan *et al.*, 2016), classify and differentiate existing motifs (Duarte *et al.*, 2003) and reveal conformational changes in related structures (Duarte *et al.*, 2003; Giambaşu *et al.*, 2010; Zhao *et al.*, 2015). Similar to the PROCHECK program (Laskowski *et al.*, 1993) for protein structures, it has also been used to assess new structures for unusual or potentially ‘disallowed’ regions (Lakomek *et al.*, 2010; Montemayor *et al.*, 2014; Ren *et al.*, 2017, 2021).

Several programs have been developed to analyze RNA structure utilizing the eta/theta formalism. The Perl programs AMIGOS (Duarte and Pyle, 1998) and PRIMOS (Duarte *et al.*, 2003) calculate eta/theta angles for RNA and perform RNA motif searching by first distilling 3D structures into a linearized set of eta/theta angles known as RNA worms. RNA or DNA worms provide computationally searchable and comparable roadmaps of 3-D structure by describing each nucleotide as a function of its sequence position and eta/theta angles (Duarte *et al.*, 2003). The Java program AMIGOS II (Wadley *et al.*, 2007) combines the functionality of AMIGOS and PRIMOS in a single graphical user interface application.

Despite its utility, AMIGOS II has several shortcomings. First, AMIGOS II lacks the ability to parse DNA structures—a feature which would enable the analysis of unusual DNA structures, such as DNA aptamers. Second, AMIGOS II is unable to calculate etaʹ (C1ʹ_*i*__−__1_–P_*i*_–C1ʹ_*i*_–P_*i*__+1_) and thetaʹ (P_*i*_–C1ʹ_*i*_–P_*i*__+1_–C1ʹ_*i*__+1_) angles (Gruene and Sheldrick, 2011; Keating and Pyle, 2010), which are easier to accurately determine from experimental density maps than eta and theta angles. Here, we describe the reimplementation of AMIGOS II as AMIGOS III. AMIGOS III is written in Python and functions as a plugin for the popular molecular graphics system PyMOL (Schrodinger, 2021). AMIGOS III is compatible with PyMOL versions ≥2.5 and may be used with Windows, macOS, and Linux. AMIGOS III not only offers the major features of AMIGOS and PRIMOS, but it also expands their use to DNA, introduces nucleic acid worm visualization and etaʹ/thetaʹ visualization, and provides easier probe worm selection for motif searching and faster worm database generation.

## 2 Overview of AMIGOS III

AMIGOS III has two major features: NARama and motif searching. Both features can be used to analyze RNA and DNA structures, as long as the structures have defined coordinates for C4ʹ and P atoms for eta/theta calculation or C1ʹ and P atoms for etaʹ/thetaʹ calculation. Therefore, AMIGOS III can work with structures from coarse-grained simulations such as those from SimRNA (Boniecki *et al.*, 2016) and Vfold3D (Xu *et al.*, 2014).

### 2.1 NARama

**Fig. 1.**
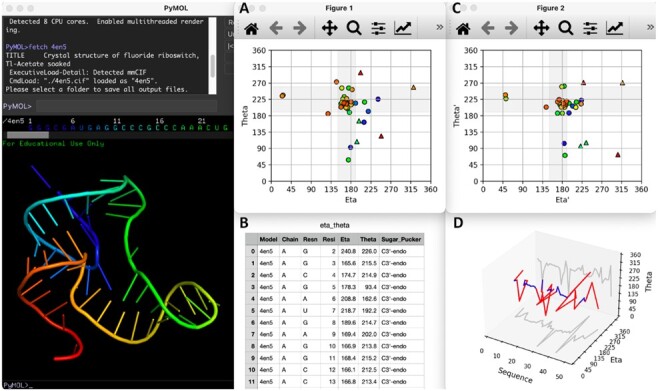
Overview of the NARama feature of AMIGOS III, showing (**A**) an eta/theta plot, (**B**) its corresponding spreadsheet, (**C**) an etaʹ/thetaʹ plot, and (**D**) a nucleic acid worm plot (helical regions in blue; non-helical regions in red) for a fluoride riboswitch (PDB 4EN5). The plots and spreadsheet shown were generated by loading the structure into PyMOL, selecting ‘NARama’ under the plugin menu, and following the prompts to select the output directory and desired plot types

The NARama feature of AMIGOS III recreates the functionality of AMIGOS with improved visualization and introduces visualization of nucleic acid worms. The NARama feature allows users to generate 2-D eta/theta and etaʹ/thetaʹ plots and 3-D nucleic acid worm plots (Fig. 1) using Matplotlib. On the 2-D plots, the color of the point matches the color of the nucleotide in the PyMOL session, and the shape of the point denotes the sugar pucker of the nucleotide (circle for C3ʹ-endo; triangle for C2ʹ-endo) (Supplementary Text S1), providing enhanced visualization compared to AMIGOS II. The plotting of etaʹ/thetaʹ is also a new feature to AMIGOS III compared to previous versions of the program. The 3-D plot displays the (eta, theta) coordinates of all nucleotides in the input selection as a function of their position in the sequence, offering the first built-in visualization of nucleic acid worms. Helical regions are shown in blue and non-helical regions are shown in red, allowing for rapid motif characterization and discovery.

### 2.2 Motif searching

The motif searching feature of AMIGOS III reimplements PRIMOS by allowing users to generate worm databases and to perform worm searches. Each worm search conducts structural comparisons between a probe worm and equivalent length worms from a worm database using eta/theta formalism. In 59.8% of the time required by PRIMOS (Supplementary Table S1), AMIGOS III can generate a worm database by creating a CSV file of eta and theta angles for each nucleic acid chain in an input directory. To perform a worm search, AMIGOS III prompts the user to select the directory containing the worm database and to select a probe worm as either a PyMOL object or a local file. The ability to select a probe worm directly from the structure loaded in the PyMOL session allows for more streamlined and user-friendly motif searching within minutes (Supplementary Fig. S1). AMIGOS III outputs a single text file containing the results from the motif search, using the same scoring methods as PRIMOS and AMIGOS II (Supplementary Text S2).

## 3 Results

As a case study, AMIGOS III was used to analyze the structure of a fluoride riboswitch (PDB 4EN5) (Fig. 1) and a modified DNA aptamer (PDB 7MK1) (Supplementary Fig. S2). The NARama feature provides a variety of structural observations, including which nucleotides adopt different sugar pucker conformations and which regions have non-helical character.

## Data availability statement

The data underlying this work are available at https://doi.org/10.6084/m9.figshare.19552441.

## Funding

This work was supported by the Gruber Science Foundation (to M.S.); the Howard Hughes Medical Institute (to C.Z. and A.M.P.); and the National Human Genome Research Institute [HG011868 to A.M.P.].

*Conflict of Interest*: none declared.

## Supplementary Material

btac207_Supplementary_Data
