# Supplementary material for: AMIGOS III: pseudo-torsion angle visualization and motif-based structure comparison of nucleic acids
Source: Bioinformatics. 2022 Apr 6;38(10):2937–9. doi: 10.1093/bioinformatics/btac207 (PMC9113296; doi:10.1093/bioinformatics/btac207)
Supplement: btac207_Supplementary_Data [file btac207_supplementary_data.docx]

**Supplementary information**

**Text S1.** AMIGOS III determines the sugar pucker of a nucleotide by calculating the torsion angle formed by atoms C1’, C2’, C3’, and C4’ (Altona and Sundaralingam, 1972). If the torsion angle is greater than 180º, the sugar pucker is classified as C2’-endo. If the torsion angle is less than 180º, it is classified as C3’-endo. On the two-dimensional plots of eta/theta and eta’/theta’, nucleotides that exhibit the C2’-endo conformation appear as triangles and nucleotides that exhibit the C3’-endo conformation appear as circles.

**Text S2.** AMIGOS III uses the same scoring methods for motif searching as PRIMOS (Duarte et al., 2003) and AMIGOS II (Wadley et al., 2007). For two nucleic acid worms of the same length, the difference in their eta/theta values at each nucleotide position, ${\Delta(\eta,\theta)}_{i}$, can be calculated using the following formula where i is the nucleotide position and A and B are the structures being compared:

${\Delta(\eta,\theta)}_{i}\equiv\sqrt{\left( \eta_{i}^{A}-\eta_{i}^{B} \right)^{2}+\left( \theta_{i}^{A}-\theta_{i}^{B} \right)^{2}}$ **(1)**

The above formula is modified if either of the following conditions are true:

$X=\left| \eta_{i}^{A}-\eta_{i}^{B} \right|>180^{\circ} or X=\left| \theta_{i}^{A}-\theta_{i}^{B} \right|>180^{\circ}$ **(2)**

In such cases, the quantity $(360^{\circ}-X)$is substituted for $(\eta_{i}^{A}-\eta_{i}^{B})$ in the original formula **1**, thus accounting for the fact that the edges of the eta/theta plot are contiguous. An overall score, $\overline{\Delta(\eta,\theta)}$, is also calculated using the following formula where n is the total number of nucleotides in the probe worm:

$\overline{\Delta(\eta,\theta)}=\frac{\sum_{i=1}^{n} {\Delta(\eta,\theta)}_{i}}{n}$ **(3)**

**Table S1.** CPU time required for AMIGOS III and PRIMOS to generate a worm database from an input directory containing 1,290 RNA PDB files. All computation was performed on Yale’s Farnam cluster.

| **Program** | **CPU Time (s)** |
| --- | --- |
| PRIMOS | 107 |
| AMIGOS III | 64 |

**Figure S1.** CPU time required for AMIGOS III to perform motif searches with probe worms of various length and the worm database generated in Table S1. All computation was performed on Yale’s Farnam cluster.


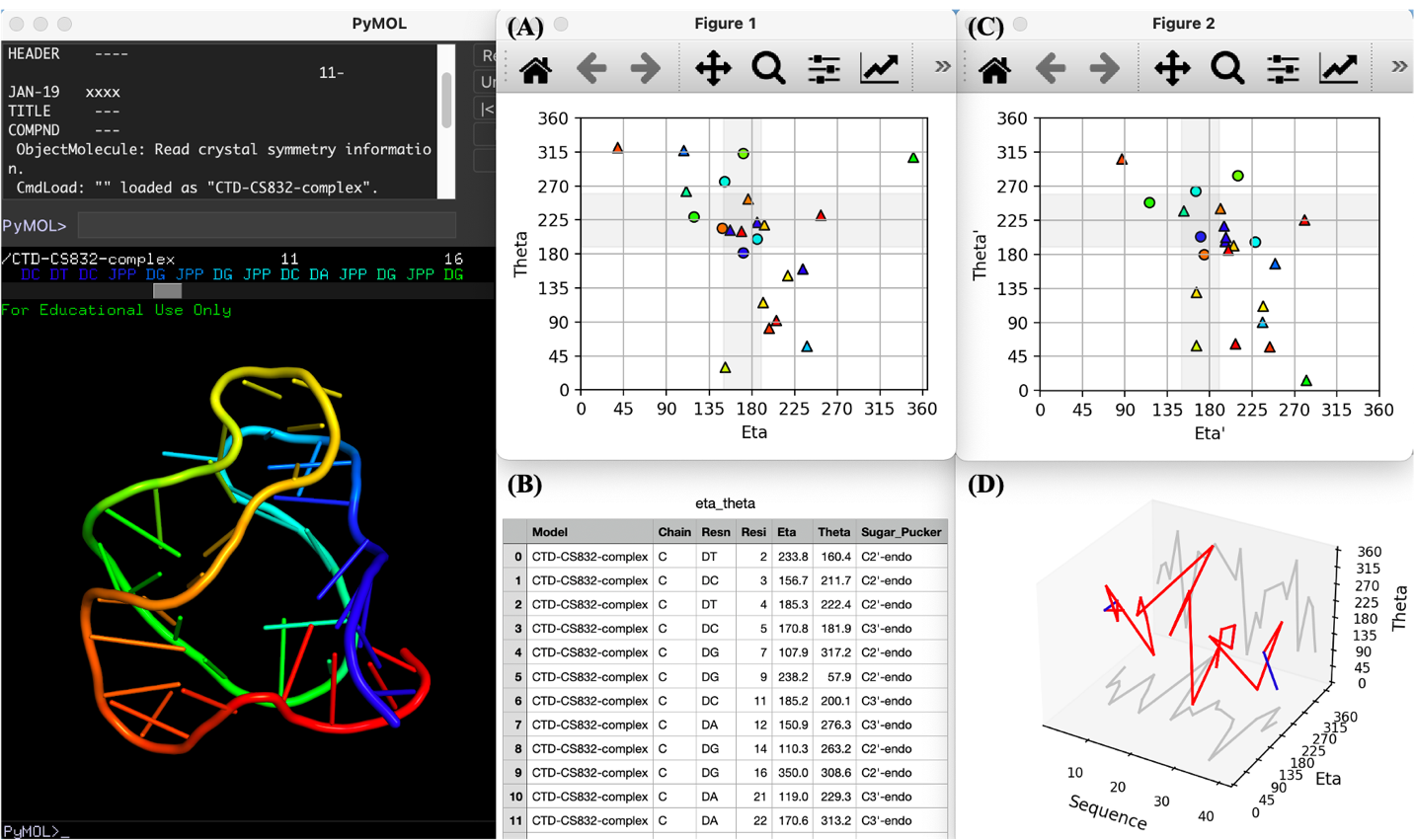


**Figure S2.** Outputs from the NARama feature of AMIGOS III for a modified DNA aptamer (PDB 7MK1, chain C), including **(A)** an eta/theta plot, **(B)** its corresponding spreadsheet, **(C)** an eta’/theta’ plot, and **(D)** a nucleic acid worm plot (helical regions in blue; non-helical regions in red). This demonstrates that AMIGOS III can be used to analyze a wide variety of structures, including DNA molecules with highly unusual backbone geometries.

References

Altona,C. and Sundaralingam,M. (1972) Conformational analysis of the sugar ring in nucleosides and nucleotides. New description using the concept of pseudorotation. *J. Am. Chem. Soc*., **94**, 8205-8212.

Duarte,C.M. et al. (2003) RNA structure comparison, motif search and discovery using a reduced representation of RNA conformational space. *Nucleic Acids Res*., **31**, 4755-4761.

Wadley,L.M. et al. (2007) Evaluating and learning from RNA pseudotorsional space: Quantitative validation of a reduced representation for RNA structure. *J. Mol. Biol*., **372**, 942-957.
